# Editorial Comment: Interaction between the impact of the Coronavirus disease 2019 pandemic and demographic characteristics on sexual/erectile dysfunction in Latin America: crosssectional study

**DOI:** 10.1590/S1677-5538.IBJU.2021.764.1

**Published:** 2022-04-14

**Authors:** Valter Javaroni

**Affiliations:** 1 Hospital Federal do Andaraí Rio de Janeiro Departamento de Andrologia Rio de Janeiro RJ Brasil Departamento de Andrologia, Hospital Federal do Andaraí Rio de Janeiro, Rio de Janeiro, RJ, Brasil

## COMMENT

The global outbreak of coronavirus disease (COVID-19) caused by the severe acute respiratory syndrome coronavirus 2 (SARS-CoV-2) the consequent lockdown had dramatic repercussions at both macrosocial, such as the economy and policy, and microsocial level, such as on the psychological and relational well-being of persons represents an unprecedented challenge for healthcare (1).

In June 2020, more than 7.5 million COVID-19 cases have been confirmed worldwide, with more than 420,000 lives lost due to the disease (2), and increased rates of post-traumatic stress disorder (PTSD), depression and anxiety were already expected in the general population, and even more in COVID-19 survivors, following the pandemic (3-6).

It has already been shown that sexual dysfunction may be observed in PTSD patients (7), and that emotional numbness can prevent emotional intimacy and connectedness with a partner. So, PTSD symptoms can cause problems in sexual functions, and PTSD can be used as a predictive parameter for sexual dysfunction in these patients (8). The high level of anxiety, anger and irritability observed in PTSD patients not only creates sexual dysfunction independently but also affects sexual dysfunction indirectly due to negative effects on social or romantic relationships and intimacy with the opposite sex (9). It can worse preexisting dysfunctions. Moreover, anger and anxiety might have a bidirectional relationship with erectile function. That is, anger and anxiety can create erectile dysfunction, and sexual dysfunction can induce or increase these symptoms (10).

The relationship between erection dysfunction and psychological state has also been examined in large-scale studies. In the National Health and Social Life Survey (NHSLS) study, data show that emotional problems and stress-related problems pose a risk of difficulty being experienced at all stages of sexuality. The researchers concluded that psychological state was an independent factor affecting sexual function (11).

In ‘The multinational men's attitudes to life events and sexuality’ (MALES) study conducted in Europe and North and South America, which included 2,912 men, depression or anxiety was found in 25% of the patients who reported to have ED, while was found in 26% of the patients who reported to have depression or anxiety (12).

So, as sexual activity is closely associated with mental and psychological health, it is unsurprising that sexual desire and frequency have declined in both genders during this pandemic (13, 14).

An interesting paper from Spain suggested that the social impact of the lockdown is related to gender, age and socioeconomic conditions. Authors found that women and young people had worse mental health outcomes during lockdown (15).

Another Italian paper confirmed that COVID-19 lockdown dramatically impacted on psychological, relational, and sexual health of the population and found that sexual activity played a protective effect, in both genders (16).

Chinese authors conducted a cross-sectional survey using an online questionnaire applied in young individuals (15-35 years old) and found that many young people had decreased sexual desire and frequency of sexual intercourse due to COVID-19. In addition, a relatively large number of participants reported a significant reduction in alcohol-related sexual consequences and risky sexual behavior, an increase in masturbation and in the use of pornography. They speculate that increased family supervision or interference, less personal freedom overall, and poor mental health and partner relationships are likely contributors to these changes in sexual behavior (17).

Evaluating healthcare professionals, Bulut, et al. found that during the COVID-19 outbreak, healthcare professionals are exposed to psychological trauma and their sexual function was negatively affected (18).

Focusing on the interaction between the impact of the Coronavirus disease 2019 pandemic and demographic characteristics on sexual/erectile dysfunction in Latin America, we could make some considerations.

First is essential to recognize the importance, during a pandemic, to obtain customized data that point out the peculiarities of each affected region, allowing more effective preventive actions. But it's also known that management of sexual difficulties often requires addressing complex causes rooted in psychological, relational, and sociocultural spheres (19)

Available pre-pandemic data suggest a wide range for the prevalence of sexual dysfunction, which is influenced by ascertainment methods, age of the participants, presence or absence of various physical and psychiatric comorbidities (20). In general, it is suggested that about 43% of women and 31% of men have one or other kinds of sexual dysfunction, with premature ejaculation being the most common sexual dysfunction occurring in males and hypoactive sexual desire disorder in females (21).

Since we do not have a pre-pandemic photography of prevalence among the sample analyzed in this study nor a control group, it is hard to see the real magnitude of this interaction proposed by authors (22).

In terms of Latin America specific population characteristics, the educational level and family income of the analyzed group seems quite higher than the media of the habitants of those low and middle income countries.

As authors recognized, widely used “gold standard” instruments such as the International Index of Erectile Function (23) and the Female Sexual Function Index (24-25), are too narrowly focused. For instance, both assume vaginal penetration and have limited questions on the relationship or on patient assessment of “bother.”

On the other hand, as occurs when using epidemiological tools, the main difficult is to deal with the challenge of distinguishing mild difficulties found in questionaries scores from clinical sexual dysfunction (26). Mild and transient sexual function problems are sufficiently common (27) to be considered “normal”.

In the fifth edition of The Diagnostic and Statistical Manual of Mental Disorders (DSM-5), two new conditions for morbidity were added to the existing distress criterion stipulated in DSM-IVtr. There is now a requirement, across all diagnoses, that symptoms have persisted for a minimum duration of approximately six months; have been experienced in almost all or all (approximately 75%-100%) sexual encounters or have been persistent/recurrent; and have caused the individual clinically significant distress. The changes were specifically designed to improve precision, “reduce likelihood of overdiagnosis” and “distinguish transient sexual difficulties from more persistent sexual dysfunction” (28).

Similar to the findings of the authors who identified a greater pandemic “impact over the erectile/sexual function of people who lived with a partner” a chinese study during SARS epidemic in 2009, erectile dysfunction rates were also higher in married men but with a different explanation: they believe this was related to the mean age of the married patient group being high and the anxiety they experienced in relation to their families being greater. It was stated that married men were more concerned about their own health and that of their families, especially their children, in some studies conducted after the SARS epidemic, and it was therefore concluded that married people showed more PTSD symptoms (29).

Finally, it is not feasible (and certainly not the aim of the manuscript) for cross-sectional surveys to provide sufficient clinical information for a definite diagnosis. For instance, the already mentioned DSM-5 stipulates that if the sexual problem is attributable to a medical condition (in this case PSTD), then a diagnosis of sexual dysfunction is not given; and off course it is even not possible to ascertain such causality in a cross-sectional survey (26).

Adding this evidence to the existing ones alerting to the negative interference of the pandemic on the mental and sexual health of the population, it is urgent to disseminate qualified information and invest in the adequate preparation of health professionals to address issues that may not be actively presented during anamnesis.

Faced with the congestion of health services generated by the pandemic and the necessary prioritization of critical care, doors were closed even for those who were trying to provide care to deal with dysfunctions. In countries where the needy population was already facing difficulties, the impact of the pandemic certainly cannot yet be full scaled (30).

Several recommendations were made on policies. For instance: the use of telemedicine and community-based programs as a way to deliver sexual services during and after a pandemic (31). The determination of the groups most vulnerable is important for the planning of training and action of psychological support teams. The fight against pandemics should include health teams with strong psychological grounding, to offer qualified medical care for patients (32). And successful use of quarantine as a public health measure requires to reduce, as far as possible, the negative effects associated with it. Officials should take every measure to ensure that this experience is as tolerable as possible for people. This can be achieved as suggested by Brooks et al. by: telling people what is happening and why, explaining how long it will continue, providing meaningful activities for them to do while in quarantine, providing clear communication, ensuring basic supplies (such as food, water, and medical supplies) are available, and reinforcing the sense of altruism that people should, rightly, be feeling (1).
